# Characterizing e-Cigarette–Related Videos on TikTok: Observational Study

**DOI:** 10.2196/42346

**Published:** 2023-04-05

**Authors:** Zidian Xie, Siyu Xue, Yankun Gao, Dongmei Li

**Affiliations:** 1 Department of Clinical and Translational Research University of Rochester Medical Center Rochester, NY United States; 2 Goergen Institute for Data Science University of Rochester Rochester, NY United States

**Keywords:** e-cigarette, TikTok, video, provaping, antivaping

## Abstract

**Background:**

As a popular social networking platform for sharing short videos, TikTok has been widely used for sharing e-cigarettes or vaping-related videos, especially among the youth.

**Objective:**

This study aims to characterize e-cigarette or vaping-related videos and their user engagement on TikTok through descriptive analysis.

**Methods:**

From TikTok, a total of 417 short videos, posted between October 4, 2018, and February 27, 2021, were collected using e-cigarette or vaping-related hashtags. Two human coders independently hand-coded the video category and the attitude toward vaping (provaping or antivaping) for each vaping-related video. The social media user engagement measures (eg, the comment count, like count, and share count) for each video category were compared within provaping and antivaping groups. The user accounts posting these videos were also characterized.

**Results:**

Among 417 vaping-related TikTok videos, 387 (92.8%) were provaping, and 30 (7.2%) were antivaping videos. Among provaping TikTok videos, the most popular category is vaping tricks (n=107, 27.65%), followed by advertisement (n=85, 21.95%), customization (n=75, 19.38%), TikTok trend (n=70, 18.09%), others (n=44, 11.37%), and education (n=6, 1.55%). By comparison, videos showing the TikTok trend had significantly higher user engagement (like count per video) than other provaping videos. Antivaping videos included 15 (50%) videos with the TikTok trend, 10 (33.33%) videos on education, and 5 (16.67%) videos about others. Videos with education have a significantly lower number of likes than other antivaping videos. Most TikTok users posting vaping-related videos are personal accounts (119/203, 58.62%).

**Conclusions:**

Vaping-related TikTok videos are dominated by provaping videos focusing on vaping tricks, advertisement, customization, and TikTok trend. Videos with the TikTok trend have higher user engagement than other video categories. Our findings provide important information on vaping-related videos shared on TikTok and their user engagement levels, which might provide valuable guidance on future policy making, such as possible restrictions on provaping videos posted on TikTok, as well as how to effectively communicate with the public about the potential health risks of vaping.

## Introduction

Since entering the market in 2003, the use of e-cigarettes (vaping) has increased rapidly worldwide, especially among US middle school and high school students [1]. In the United States, the percentage of high school e-cigarette users has increased from 1.5% in 2011 to 27.5% in 2019, and the percentage among middle school students has changed from 0.6% to 10.5% in this period [2,3]. There were still 1.72 million US high school students and 320,000 middle school students who reported e-cigarette use in 2021 [4]. Although the long-term health effects of vaping remain unknown, vaping was associated with various health risks, including respiratory disorders (eg, wheezing and chronic obstructive pulmonary disease), cardiovascular diseases, mental health problems, and even cancer [5-10]. Therefore, it is of utmost importance to alleviate this vaping epidemic to protect public health, especially among the youth.

With the increasing prevalence of social media use especially among the youth, the impact of social media exposure (eg, YouTube, Twitter, and Instagram) on user behaviors is significant [11]. Previous studies showed that exposure to cigarette advertising and marketing was associated with increased smoking levels among adolescents [12,13]. To promote their e-cigarette products, e-cigarette companies and vape stores have been using social media platforms, such as YouTube and Instagram, for marketing [14,15]. Studies showed that provaping content is dominant on social media, such as Instagram and YouTube [16,17]. Previous studies showed that higher social media e-cigarette exposure is associated with a higher likelihood of current and future e-cigarette use, especially among adolescents [18-20]. The visual imagery of vaping has been proven to be the cue for the desire for regular cigarette and e-cigarette use [21].

It reported that 21% of American adults claimed they had ever used TikTok, and 55% of 18- to 29-year-olds said they had used TikTok in 2021 [22]. In 2022, it was estimated that about 20% of TikTok users are younger than 18 years in the United States [23]. Originally designed for creating and sharing short videos, TikTok has been used by e-cigarette companies and vape stores to promote e-cigarette products and other related products. The promotion formats included direct marketing and sponsoring certain users to post vaping-related videos. TikTok has become a popular channel for users to post e-cigarette or vaping-related videos, which leads to high user engagement [24-26]. However, more in-depth content analysis on vaping-related TikTok videos, especially their user engagement, is limited.

This study aimed to characterize the content of vaping-related short videos on TikTok by hand-coding and classifing them into different video categories. The user engagement levels (eg, the number of likes, comments, shares, and plays) of vaping-related TikTok videos between different video categories were further compared. In addition, the characteristics of TikTok user accounts that posted these vaping-related videos were examined. Our findings would provide valuable information for public health entities to understand the vaping-related content shared on TikTok and, more importantly, develop an effective antivaping communication approach to alleviate the vaping epidemic, especially among the youth.

## Methods

### Data Collection

TikTok short videos (7-60 seconds) were collected through a TikTok application programming interface wrapper using 120 e-cigarette or vaping-related hashtags (Multimedia Appendix 1), such as “vaping,” “vapes,” and “ecig” [25]. The 500 unique videos were collected on February 27, 2021. The posted dates of these videos range from October 4, 2018, to February 27, 2021. The relevance of videos was determined by checking if the video content was related to vaping or e-cigarettes. Out of 500 videos, we identified a total of 417 videos related to vaping or e-cigarettes. The rest of the 83 videos were considered not relevant since these videos were not showing vaping or e-cigarette–related content, such as videos showing people pretending to vape but playing instruments or young people talking, singing, or dancing, etc.

To determine the user engagement levels of these e-cigarette or vaping-related videos, the metadata for 417 TikTok videos related to e-cigarettes or vaping, including the comment count, like count, share count, and play count, were collected. To study the TikTok users who posted these videos, we manually collected the user account information about 203 unique TikTok users who posted these 417 videos, including the follower count (the number of TikTok users following this account), following count (the number of user accounts that this TikTok user is following), and video count (the number of videos posted by this user account).

### Ethics Approval

The study has been reviewed and approved by the Office for Human Subject Protection Research Subjects Review Board at the University of Rochester (Study ID: STUDY00006570).

### Data Coding

To characterize these TikTok videos, we randomly selected 100 TikTok videos related to vaping or e-cigarettes from our collected videos. Then, 2 individual human coders (YG and SX) carefully watched each video independently and further labeled the attitude of each video toward vaping or e-cigarettes and the video category based on the video content. The codebook was developed through group discussion (ZX, YG, SX, and DL; Multimedia Appendix 2). We labeled the attitude of each video as either provaping or antivaping. A video was labeled as “provaping” if it supported e-cigarette use or vaping, such as showing a vaping scene, e-cigarette promotion, e-cigarette customization, or argument for the benefits of vaping. A video was classified as “antivaping” if it discouraged e-cigarette use or vaping, such as listing potential health risks with vaping.

We categorized each provaping video into six video categories based on previous studies [17,24].

1. “Advertisement”: videos containing store promotion and product display (displaying product brand in the video)
2. “Customization”: videos showing the customization process of e-cigarettes, including changing the coil and teaching how to use the e-cigarettes
3. “Education”: videos providing information on the benefits of vaping
4. “TikTok trend”: videos following TikTok trends, including the “Vape? No xxx” trend and “vape and hold breath” challenge
5. “Vaping trick”: videos performing vaping tricks
6. “Others”: videos do not belong to the above categories but with a small sample size

In addition, we categorized antivaping videos into three video categories, including

1. “TikTok trend”: videos showing vaping cessation trends or challenges
2. “Education”: videos showing potential health risks associated with vaping
3. “Others”: videos do not belong to the above categories but do not have enough samples to make its own category, such as joking about vaping while playing video games

TikTok user accounts that posted vaping-related videos have been classified into five categories based on their characteristics (Multimedia Appendix 3).

1. “Sponsored”: user profiles or videos clearly indicating the sponsorship
2. “Vape store”: users working in vaping stores or official account from the vaping stores and their accounts mainly post vaping-related videos shot in the store
3. “Influencer”: TikTok user account with the TikTok blue checkmark indicating they are influencers
4. “Business organization”: users posting vaping-related videos but the account mainly promotes other businesses unrelated to vaping
5. “Personal”: individual TikTok users posting vaping-related videos for sharing their vaping experiences

All videos and TikTok user accounts were double-coded by 2 coders (YG and SX) based on the codebooks. The agreement between the 2 independent coders reached 98.5% for TikTok user account types, and the κ statistics for interrater reliability is 0.957 for video coding. All discrepancies between the 2 human coders were resolved through discussion within a group of 4 members (ZX, YG, SX, and DL).

### Statistical Analysis

Considering the different number of TikTok videos in each category and the skewed distribution of the user engagement measures, basic statistics (median, first quartile [Q1], and third quartile [Q3]) on the user engagement measures (including the number of comments, likes, plays, and shares) for each video category were calculated. In addition, the median, Q1, and Q3 were calculated on the number of followers, followings, and vaping-related videos posted by each TikTok user account. Negative binomial regression models were used to compare the average number of comments, likes, and shares between different video categories for provaping and antivaping by controlling the number of plays for each video. Pairwise comparisons of the number of comments, likes, and shares among video categories were conducted using the “Tukey” method for the multiplicity adjustment. All statistical analyses were conducted using the statistical analysis software R (2017; R Foundation for Statistical Computing).

## Results

### Characteristics of Vaping-Related TikTok Videos

**Figure 1:**
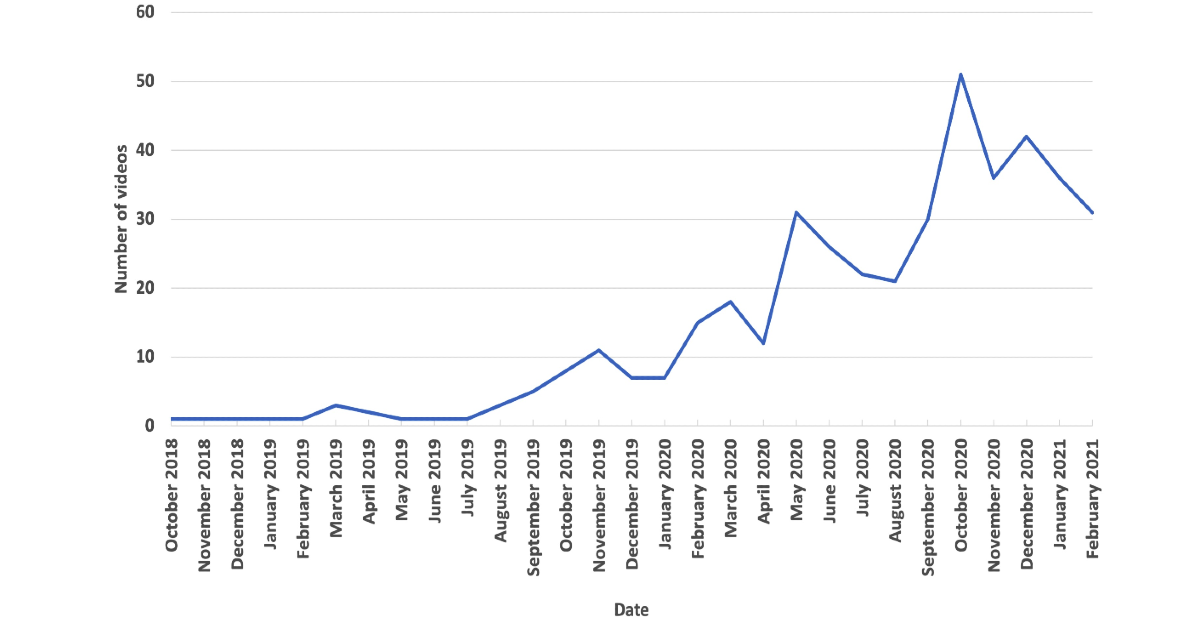
Number of vaping-related TikTok videos over time.

Among 500 TikTok videos collected using vaping-related hashtags, there were 417 videos related to e-cigarette or vaping based on our hand-coding results. Among them, 387 (92.8%) were provaping, and 30 (7.2%) were antivaping. As shown in Figure 1, the number of vaping-related TikTok videos has been increasing significantly since October 2018 and had a peak in October 2020 with a slight decrease afterward.

There were 3 video categories for antivaping videos, including 15 videos about the TikTok trend, 10 videos on education, and 5 videos about others (Table 1). Among provaping TikTok videos, the most popular category was vaping tricks (107/387, 27.65%), followed by advertisement (85/387, 21.95%), customization (75/387, 19.38%), TikTok trend (70/387, 18.09%), others (44/387, 11.37%), and education (6/387, 1.55%).

**Table 1:** Characteristics of vaping-related TikTok videos.

Attitude and category			Videos, n (%)		Likes (n), median (Q1^a^, Q3^b^)		Comments (n), median (Q1, Q3)		Shares (n), median (Q1, Q3)		Plays (n), median (Q1, Q3)
**Provaping**											
	Vaping trick	107 (27.65)		7363 (2411, 21,050)		58 (30, 161)		75 (15, 237)		67,500 (23,950, 168,700)	
	Advertisement	85 (21.96)		6291 (2127, 22,400)		83 (25, 181)		61 (9, 162)		78,800 (29,300, 166,500)	
	Customization	75 (19.38)		12,500 (3369, 41,950)		43 (15, 156)		43 (11, 150)		230,900 (43,450, 745,150)	
	TikTok trend	70 (18.09)		17,350 (5504, 117,750)		84 (29, 403)		57 (16, 396)		84,550 (47,150, 599,450)	
	Others	44 (11.37)		2717 (613, 13,000)		52 (10, 115)		16 (4, 60)		27,400 (8154, 79,825)	
	Education	6 (1.55)		11,400 (3743, 15,250)		72 (31, 215)		91 (31, 111)		71,750 (27,650, 78,200)	
	Total	387		8146 (2494, 29,000)		62 (21, 182)		49 (12, 182)		73,900 (26,150, 323,900)	
**Antivaping**											
	TikTok trend	15 (50.00)		29,000 (17,200, 114,050)		173 (118, 574)		51 (19, 258)		163,700 (106,550, 709,400)	
	Education	10 (33.33)		7403 (2746, 19,925)		127 (113, 267)		329 (21, 1338)		75,550 (34,700, 289,050)	
	Others	5 (16.67)		58,900 (15,200, 108,400)		637 (237, 1433)		451 (225, 1245)		252,100 (175,000, 366,400)	
	Total	30		21,400 (12,725, 96,725)		190 (116, 773)		98 (19, 1104)		169,350 (73,375, 408,275)	

^a^Q1: first quartile.

^b^Q3: third quartile.

### User Engagement of Vaping-Related TikTok Videos

By comparison, in general, the user engagement in antivaping TikTok videos is higher than that of provaping videos. As shown in Table 1, the median number of likes for antivaping videos is 21,400 likes per video, while it is 8146 likes per video for provaping videos. Similarly, the median number of comments (190 vs 62), shares (98 vs 49), and plays (169,350 vs 73,900) for antivaping videos are higher than that for provaping videos.

As shown in Table 1 and Multimedia Appendix 4, among provaping videos, videos showing the TikTok trend have the highest median number of likes (17,350 likes per video) and comments (84 comments per video). Provaping videos with education have a higher median number of shares (91 shares per video) than other provaping videos. In addition, videos with customization have the highest median number of plays (230,900 plays per video) among provaping videos. Among antivaping videos shown in Table 1 and Multimedia Appendix 5, videos in others have the highest user engagement, including the median number of likes (58,900 likes per video), comments (637 comments per video), shares (451 shares per video), and plays (252,100 plays per video).

Considering the potential impact of the number of plays on the user engagement (likes, comments, and shares) for each video and to better compare the differences in the user engagement measures between different vaping-related TikTok videos, we performed a negative binomial analysis by controlling for the number of plays of each video. As shown in Table 2, videos with the TikTok trend have a significantly higher number of likes than other provaping video types (including vaping tricks, advertisement, customization, and others) with a *P*<.001. In addition, provaping videos with customization have a significantly lower number of likes than most other categories, such as vaping tricks, advertisement, TikTok trend, and others. Compared to other provaping videos, videos with customization have a significantly lower number of comments and shares. Among antivaping videos, videos with education have a significantly lower number of likes than other categories (*P*=.02), but not on the number of shares and comments (Multimedia Appendix 6).

**Table 2:** Different user engagement levels among provaping TikTok videos.

Category comparison	Comments		Likes		Shares	
	Estimate (SE)	*P* value	Estimate (SE)	*P* value	Estimate (SE)	*P* value
Advertisement vs others	0.247 (0.189)	.76	−0.067 (0.096)	.98	0.267 (0.212)	.79
Customization vs others	−1.037 (0.199)	<.001	−0.439 (0.102)	<.001	−1.184 (0.222)	<.001
Education vs others	0.323 (0.433)	.97	0.105 (0.223)	>.99	0.290 (0.476)	.99
TikTok trend vs others	0.329 (0.198)	.53	0.401 (0.101)	<.001	0.090 (0.221)	>.99
Vaping trick vs others	−0.009 (0.183)	>.99	0.017 (0.093)	>.99	0.172 (0.204)	.96
Customization vs advertisement	−1.284 (0.162)	<.001	−0.371 (0.083)	<.001	−1.451(0.178)	<.001
Education vs advertisement	0.076 (0.419)	>.99	0.172 (0.216)	.96	0.023 (0.460)	>.99
TikTok trend vs advertisement	0.082 (0.162)	>.99	0.468 (0.083)	<.001	−0.177 (0.179)	.91
Vaping trick vs advertisement	−0.256 (0.145)	.47	0.084 (0.075)	.86	−0.095 (0.161)	.99
Education vs customization	1.361 (0.423)	.01	0.543 (0.218)	.11	1.474 (0.463)	.02
TikTok trend vs customization	1.366 (0.166)	<.001	0.839 (0.085)	<.001	1.274 (0.183)	<.001
Vaping trick vs customization	1.028 (0.153)	<.001	0.456 (0.078)	<.001	1.356 (0.169)	<.001
TikTok trend vs education	0.005 (0.423)	>.99	0.296 (0.218)	.73	−0.200 (0.463)	>.99
Vaping trick vs education	−0.332 (0.416)	.96	−0.088 (0.215)	>.99	−0.118 (0.456)	>.99
Vaping trick vs TikTok trend	−0.338 (0.154)	.22	−0.384 (0.079)	<.001	0.082 (0.170)	>.99

### Characteristics of TikTok User Accounts Posting Vaping-Related Videos

In total, 417 vaping-related TikTok videos were examined, which were posted by 203 unique TikTok user accounts. Among them, 160 user accounts had only 1 video in our collected videos, and the rest of the user accounts had at least 2 videos in our data set (2 user accounts had 21 vaping-related videos, respectively). As shown in Table 3, among 203 TikTok user accounts, more than half of them (n=119, 58.62%) are personal accounts, followed by vape store (n=57, 28.08%), sponsored (n=13, 6.4%), other small business (n=8, 3.94%), and influencers (n=6, 2.96%). The TikTok influencer accounts had the highest median number of followers (1,950,000) and followings (765). In contrast, personal accounts had the lowest median number of followers (8667). Other small business and influencer accounts posted the highest median number of videos (1047 and 712, respectively) while personal and vape store accounts posted the least median number of videos (79 and 79, respectively).

**Table 3:** Characteristics of TikTok users who posted vaping-related videos.

Account type	Users, n (%)	Followers (n), median (Q1^a^, Q3^b^)	Followings (n), median (Q1, Q3)	Videos (n), median (Q1, Q3)
Personal	119 (58.62)	8667 (1967, 50,950)	154 (32, 737)	79 (23, 278)
Vape store	57 (28.08)	23,600 (6128, 69,500)	56 (18, 169)	79 (37, 206)
Sponsored	13 (6.4)	113,900 (54,100, 208,400)	61 (8, 82)	264 (170, 338)
Other small business	8 (3.94)	226,000 (120,000, 490,250)	587 (156, 952)	1047 (415, 1245)
Influencer	6 (2.96)	1,950,000 (1,200,000, 3,150,000)	765 (377, 923)	712 (482, 799)

^a^Q1: first quartile.

^b^Q3: third quartile.

## Discussion

### Principal Findings

In this study, we characterized 417 vaping-related videos from TikTok, which showed an increasing trend from October 2018 to February 2021. While most vaping-related TikTok videos are provaping, the user engagement (eg, the median number of likes, comments, shares, and plays) for antivaping videos was higher than that for provaping videos. Among provaping videos, the most popular video category was vaping tricks. Videos with TikTok trends had the highest number of likes and comments among provaping videos, while videos with customization had the highest number of plays. Among antivaping videos, videos with others had the highest user engagement, including the median number of likes, comments, shares, and plays. Further statistical analysis showed that videos with TikTok trends have a significantly higher number of likes than other provaping videos. While most TikTok user accounts that posted vaping-related videos were personal accounts, the influencer accounts had the highest number of followers and followings.

### Comparison With Previous Work

Our results showed that the number of vaping-related TikTok videos has been increasing in our study period, which coincides with the increasing popularity of vaping in recent years [1]. Among the TikTok videos we collected, most were provaping, which is consistent with previous findings with vaping-related TikTok videos, YouTube videos, and Instagram posts [16,17,24,25]. Since many social media users are of younger generations, the youth is one of the most susceptible groups to be exposed to vaping-related short videos on TikTok, which might potentially affect their perceptions of vaping. The dominance of provaping posts on social media (including TikTok, YouTube, and Instagram) might partially contribute to the vaping epidemic in recent years, especially among the youth. Therefore, to alleviate the vaping epidemic among the youth, the regulation of provaping videos (especially the advertisement and showing of e-cigarette products) on TikTok should be considered.

User engagement measures (eg, the number of likes, comments, shares, and plays for each video) can reflect the popularity of TikTok videos. In this study, we showed that antivaping videos have higher user engagement than provaping videos. However, on YouTube and Instagram, antivaping videos or posts have lower user engagement than provaping [16,17]. These differences could be due to different social media platforms, and the demographics of their users are different. Nevertheless, this finding might suggest that TikTok could be a potentially effective communication channel for delivering antivaping messages, which should be considered by public health communities or organizations. Regardless of provaping or antivaping, the videos with TikTok trends have a relatively higher user engagement (eg, the number of likes, comments, and shares) than other video categories. One possible explanation is that most TikTok users are young people, and they like to participate in video challenges or trends and show their peers what they can do. Therefore, videos with vaping cessation challenges or trends should be encouraged on TikTok considering their high user engagement levels.

Among TikTok user accounts that posted vaping-related videos, more than half are personal users, and over one-quarter are from vape stores. In contrast, on Instagram, more than half of the user accounts for provaping are vape stores [16]. Therefore, while on Instagram provaping posts are more likely to be posted by vape stores, provaping videos on TikTok are more likely to be shared by personal users. One possible reason is that as a relatively new social media platform, TikTok is mainly used by individual users for sharing personal experiences but is not widely used by vape stores to promote their vaping products. However, out of 417 TikTok videos, 85 were about advertisements, which covered different e-cigarette brands, such as SMOK, HQD, and VOOPOO. Therefore, considering the prevalence of underage TikTok users, to reduce the exposure of the youth to these e-cigarette advertisements, the advertisements of e-cigarette products on TikTok need to be closely monitored and further regulated by public health authorities.

### Limitations

This study has several limitations. In this cross-sectional study, we only studied 417 TikTok videos related to vaping. Therefore, our results could have some bias due to the relatively small sample size. In addition, considering TikTok users do not represent the whole population, our findings may not be generalizable. In this study, while we have tried to use more e-cigarette or vaping-related keywords to collect relevant TikTok videos, we might miss some other relevant keywords, which could introduce some biases in our findings. Due to the restriction, the demographics (eg, age and gender) and geolocation of TikTok users who watched these vaping-related videos could not be collected. Therefore, we could not determine if there is some difference in the perception of these videos between different demographic groups. While the posted date for each vaping-related TikTok video is different, we did not control for it in our statistical analysis, which could bias our results. Lastly, the user engagement level and perception on social media may not reflect actual beliefs and behaviors [27].

### Conclusions

By examining vaping-related TikTok videos, this study characterized the features and user engagement level of both provaping and antivaping videos on TikTok, which will help public health authorities understand the nature of vaping-related TikTok videos. This study showed that the majority of vaping-related TikTok videos are provaping, which might contribute to the current vaping epidemic, especially among the youth. Therefore, these provaping posts on TikTok and other social media platforms should be especially concerning, which requires further regulation by public health authorities to protect public health. On the other hand, this study showed that antivaping videos had a relatively high user engagement on TikTok, providing another valid antivaping communication approach to effectively communicate with the public (especially the youth) about the potential health risks of vaping through social media. These antivaping TikTok videos should be encouraged and used by public health communities and campaigns. For example, considering the relatively high user engagement of antivaping videos on TikTok, more antivaping videos (especially vaping cessation challenges or trends) should be encouraged to alleviate the current vaping epidemic, especially among the youth.

## Appendix

Multimedia Appendix 1 Hashtags used to collect TikTok videos related to e-cigarettes or vaping.

Multimedia Appendix 2 Codebook for hand-coding vaping-related TikTok videos.

Multimedia Appendix 3 Codebook for hand-coding TikTok user accounts.

Multimedia Appendix 4 Boxplots of user engagement measures for different provaping categories.

Multimedia Appendix 5 Boxplots of user engagement measures for different antivaping categories.

Multimedia Appendix 6 Different user engagement levels among antivaping TikTok videos.

## Abbreviations

Q1: quartile 1
Q2: quartile 2

## Data Availability

Data are available upon reasonable request.

## References

[1] Gentzke AS, Creamer M, Cullen KA, Ambrose BK, Willis G, Jamal A, King BA (2019). Vital signs: tobacco product use among middle and high school students—United States, 2011-2018. MMWR Morb Mortal Wkly Rep.

[2] Cullen KA, Gentzke AS, Sawdey MD, Chang JT, Anic GM, Wang TW, Creamer MR, Jamal A, Ambrose BK, King BA (2019). e-cigarette use among youth in the United States, 2019. JAMA.

[3] Wang TW, Gentzke AS, Creamer MR, Cullen KA, Holder-Hayes E, Sawdey MD, Anic GM, Portnoy DB, Hu S, Homa DM, Jamal A, Neff LJ (2019). Tobacco product use and associated factors among middle and high school students—United States, 2019. MMWR Surveill Summ.

[4] Gentzke AS, Wang TW, Cornelius M, Park-Lee E, Ren C, Sawdey MD, Cullen KA, Loretan C, Jamal A, Homa DM (2022). Tobacco product use and associated factors among middle and high school students—National Youth Tobacco Survey, United States, 2021. MMWR Surveill Summ.

[5] Sleiman M, Logue JM, Montesinos VN, Russell ML, Litter MI, Gundel LA, Destaillats H (2016). Emissions from electronic cigarettes: key parameters affecting the release of harmful chemicals. Environ Sci Technol.

[6] MacDonald A, Middlekauff HR (2019). Electronic cigarettes and cardiovascular health: what do we know so far?. Vasc Health Risk Manag.

[7] Obisesan OH, Mirbolouk M, Osei AD, Orimoloye OA, Uddin SMI, Dzaye O, El Shahawy O, Al Rifai M, Bhatnagar A, Stokes A, Benjamin EJ, DeFilippis AP, Blaha MJ (2019). Association between e-cigarette use and depression in the behavioral risk factor surveillance system, 2016-2017. JAMA Netw Open.

[8] Tang MS, Wu XR, Lee HW, Xia Y, Deng FM, Moreira AL, Chen LC, Huang WC, Lepor H (2019). Electronic-cigarette smoke induces lung adenocarcinoma and bladder urothelial hyperplasia in mice. Proc Natl Acad Sci U S A.

[9] Li D, Sundar IK, McIntosh S, Ossip DJ, Goniewicz ML, O'Connor RJ, Rahman I (2020). Association of smoking and electronic cigarette use with wheezing and related respiratory symptoms in adults: cross-sectional results from the Population Assessment of Tobacco and Health (PATH) study, wave 2. Tob Control.

[10] Xie Z, Ossip DJ, Rahman I, Li D (2020). Use of electronic cigarettes and self-reported chronic obstructive pulmonary disease diagnosis in adults. Nicotine Tob Res.

[11] O'Keeffe GS, Clarke-Pearson K, Council on Communications and Media (2011). The impact of social media on children, adolescents, and families. Pediatrics.

[12] Wellman RJ, Sugarman DB, DiFranza JR, Winickoff JP (2006). The extent to which tobacco marketing and tobacco use in films contribute to children's use of tobacco: a meta-analysis. Arch Pediatr Adolesc Med.

[13] Shadel WG, Tharp-Taylor S, Fryer CS (2008). Exposure to cigarette advertising and adolescents' intentions to smoke: the moderating role of the developing self-concept. J Pediatr Psychol.

[14] Lee AS, Hart JL, Sears CG, Walker KL, Siu A, Smith C (2017). A picture is worth a thousand words: electronic cigarette content on Instagram and Pinterest. Tob Prev Cessat.

[15] Sears CG, Walker KL, Hart JL, Lee AS, Siu A, Smith C (2017). Clean, cheap, convenient: promotion of electronic cigarettes on YouTube. Tob Prev Cessat.

[16] Gao Y, Xie Z, Sun L, Xu C, Li D (2020). Electronic cigarette-related contents on Instagram: observational study and exploratory analysis. JMIR Public Health Surveill.

[17] Xie Z, Wang X, Gu Y, Li D (2021). Exploratory analysis of electronic cigarette-related videos on YouTube: observational study. Interact J Med Res.

[18] Cho H, Li W, Shen L, Cannon J (2019). Mechanisms of social media effects on attitudes toward e-cigarette use: motivations, mediators, and moderators in a national survey of adolescents. J Med Internet Res.

[19] Vogel EA, Ramo DE, Rubinstein ML, Delucchi KL, Darrow S, Costello C, Prochaska JJ (2021). Effects of social media on adolescents' willingness and intention to use e-cigarettes: an experimental investigation. Nicotine Tob Res.

[20] Pokhrel P, Fagan P, Herzog TA, Laestadius L, Buente W, Kawamoto CT, Lee HR, Unger JB (2018). Social media e-cigarette exposure and e-cigarette expectancies and use among young adults. Addict Behav.

[21] King AC, Smith LJ, Fridberg DJ, Matthews AK, McNamara PJ, Cao D (2016). Exposure to electronic nicotine delivery systems (ENDS) visual imagery increases smoking urge and desire. Psychol Addict Behav.

[22] Auxier B, Anderson M (2021). Social media use in 2021. Pew Research Center.

[23] (2022). Distribution of monthly active TikTok users in the United States as of April 2022, by age group. Statista.

[24] Basch CH, Fera J, Pellicane A, Basch CE (2021). Videos with the hashtag #vaping on TikTok and implications for informed decision-making by adolescents: descriptive study. JMIR Pediatr Parent.

[25] Sun T, Lim CCW, Chung J, Cheng B, Davidson L, Tisdale C, Leung J, Gartner CE, Connor J, Hall WD, Chan GCK (2023). Vaping on TikTok: a systematic thematic analysis. Tob Control.

[26] Morales M, Fahrion A, Watkins SL (2022). #NicotineAddictionCheck: puff bar culture, addiction apathy, and promotion of e-cigarettes on TikTok. Int J Environ Res Public Health.

[27] Hogan B, Quan-Haase A (2010). Persistence and change in social media. Bull Sci Technol Soc.

