# Supplementary material for: Characterizing e-Cigarette–Related Videos on TikTok: Observational Study
Source: JMIR Form Res. 2023 Apr 5;7:e42346. doi: 10.2196/42346 (PMC10131997; doi:10.2196/42346)
Supplement: Multimedia Appendix 1 [file formative_v7i1e42346_app1.docx]

**Multimedia Appendix 1.** Hashtags used to collect TikTok videos related to e-cigarettes or vaping.

| **Hashtags** |
| --- |
| vape |
| vaping |
| vapelife |
| e-cigarette |
| vapetricks |
| vapeshop |
| vapenation |
| vapes |
| vapetiktok |
| disposablevape |
| vapechallenge |
| vapegirl |
| vapecheck |
| vapequeen |
| vapetrick |
| vapedaily |
| vapestore |
| vapegod |
| vapeaddict |
| vapepen |
| dontvape |
| vapercommunity |
| frickvape |
| geekvape |
| vapeo |
| vapelover |
| vapeno |
| vapejuice |
| vapedly |
| vapeon |
| vapeporn |
| vapefam |
| vapecloud |
| bestvape |
| novape |
| vapetime |
| vapevideo |
| vapemod |
| mekongvape |
| vapepoland |
| vapeking |
| fakevape |
| wowvape |
| vapegirls |
| vapelyfe |
| vapememe |
| vapepolice |
| vapefyp |
| vaper |
| vapefamily |
| vapegame |
| fookvape |
| vapevillain |
| trickvape |
| vapeboy |
| liquidvape |
| vapesociety |
| vapeworld |
| vapepod |
| vapelove |
| vapesmoke |
| vapeindonesia |
| vaped |
| vapeclouds |
| newvape |
| vapegang |
| tiktokvape |
| vaperlord |
| charlievapes |
| vaperz_edge |
| vapeing |
| vapetok |
| applevapestore |
| vapemancam |
| vape_tricks |
| notvape |
| vapenah |
| vapee |
| vapesdown |
| vipvape |
| dontvapekids |
| vapevillian |
| vapersontiktok |
| popvape |
| girlswhovape |
| p2vape |
| notavape |
| vapi |
| vapenationgod |
| vapeshoplife |
| breakthevape |
| vape4life |
| vapovapo |
| savethevape |
| vapeforlife |
| vapechallge |
| wevapewevote |
| vapefamilyandfriends |
| bakevapebarbie |
| p2vapeshop |
| lifeinavapeshop |
| svapo |
| vapeindo |
| vapeisbad |
| roovape |
| vapeshopodessa |
| yousuckvapes |
| fidelvapes |
| ecig |
| ecigsmoke |
| ecigi |
| ecigmafia |
| ecigarette |
| ecigg |
| ecigs |
| ecigarett |
| ecigarettes |
| novaping |
| antivaping |
| stopvaping |
