# Supplementary material for: Characterizing e-Cigarette–Related Videos on TikTok: Observational Study
Source: JMIR Form Res. 2023 Apr 5;7:e42346. doi: 10.2196/42346 (PMC10131997; doi:10.2196/42346)
Supplement: Multimedia Appendix 2 [file formative_v7i1e42346_app2.docx]

**Multimedia Appendix 2.** Codebook for hand-coding vaping-related TikTok videos.

| **Attitude to Vaping** | **Video Category** | **Description** |
| --- | --- | --- |
| Pro-vaping  (Videos showing a vaping scene, e-cigarette promotion, e-cigarette customization, or argument for the benefits of vaping) | Advertisement | videos containing store promotion and product display (displaying product brand in the video), mainly opening a box with e-cigarette products, and showing vaping devices. |
|  | Customization | videos showing the customization process of e-cigarettes, including changing or building the coil, teaching how to use the e-cigarette devices, adding the e-liquid into the device, or charging the vape battery. |
|  | Education | videos providing more information about the benefits of vaping (for example, vaping is safer than smoking) and asking people to recycle Juul. |
|  | TikTok trend | videos following TikTok trends, including the “Vape? No xxx” trend, “vape and hold breath” challenge, and the split screen video format to show the challenge side-by-side. |
|  | Vaping trick | videos showing different vaping tricks and some tutorials for vaping tricks. |
|  | Others | videos do not belong to the above categories but do not have large samples to be individual category, for example vaping product review, opening a vape device, and showing used vape devices. |
| Anti-vaping  (Videos discouraging e-cigarette use, such as listing potential health risks with vaping) | TikTok trend | videos showing a trend of quitting vaping. |
|  | Education | videos providing more information about the potential health risks of vaping and asking people to quit vaping. |
|  | Others | Videos telling a vape joke while playing video game or testing if the lung is damaged. |
