# Supplementary material for: Characterizing e-Cigarette–Related Videos on TikTok: Observational Study
Source: JMIR Form Res. 2023 Apr 5;7:e42346. doi: 10.2196/42346 (PMC10131997; doi:10.2196/42346)
Supplement: Multimedia Appendix 3 [file formative_v7i1e42346_app3.docx]

**Multimedia Appendix 3.** Codebook for hand-coding TikTok user accounts.

| **TikTok User Account Type** | **Description** |
| --- | --- |
| Sponsored | User profiles or videos indicating sponsorship from posting vaping-related activities |
| Vape Store | Users working in vaping stores and their accounts mainly post vaping-related videos shot in the store |
| Influencer | TikTok user account with the TikTok blue checkmark |
| Business organization | Users posting some vaping-related videos, but the account mainly promotes other businesses unrelated to vaping and has direct other business links in their profiles. |
| Personal | Individual user posting vaping-related videos but not in the categories above |
