# Supplementary figures and images for: Characterizing e-Cigarette–Related Videos on TikTok: Observational Study
Source: JMIR Form Res. 2023 Apr 5;7:e42346. doi: 10.2196/42346 (PMC10131997; doi:10.2196/42346)

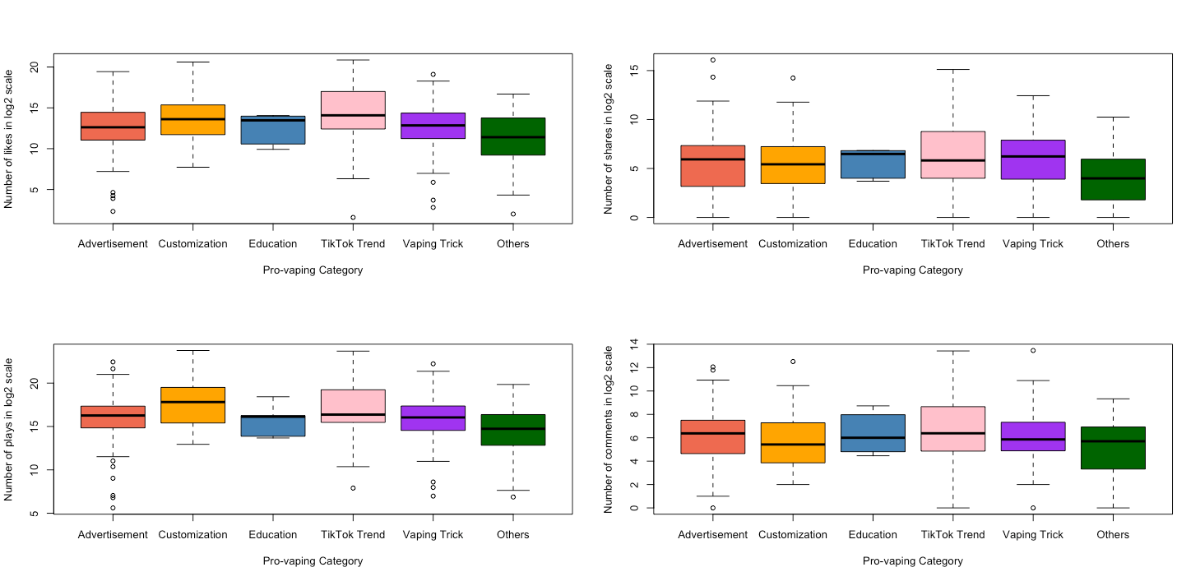

Supplement: Multimedia Appendix 4 [file formative_v7i1e42346_app4.png]

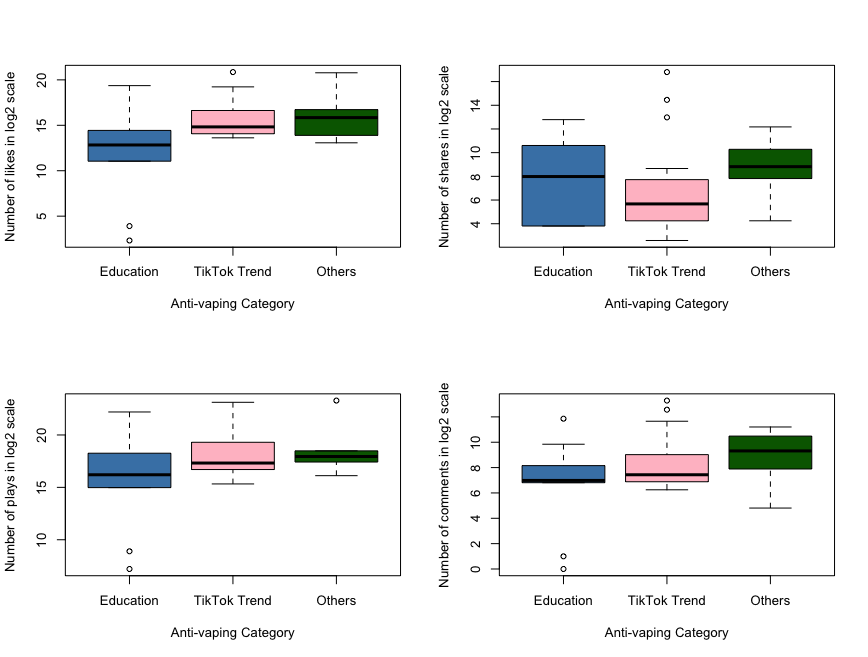

Supplement: Multimedia Appendix 5 [file formative_v7i1e42346_app5.png]
