# Supplementary material for: Characterizing e-Cigarette–Related Videos on TikTok: Observational Study
Source: JMIR Form Res. 2023 Apr 5;7:e42346. doi: 10.2196/42346 (PMC10131997; doi:10.2196/42346)
Supplement: Multimedia Appendix 6 [file formative_v7i1e42346_app6.docx]

**Multimedia Appendix 6.** Different user engagement levels among antivaping TikTok videos.

|  | **Comments** | | **Likes** | | **Shares** | |
| --- | --- | --- | --- | --- | --- | --- |
| **Category comparison** | **Estimate**  **(Std Error)** | ***P* value** | **Estimate**  **(Std Error)** | ***P* value** | **Estimate**  **(Std Error)** | ***P* value** |
| Education vs. Others | -0.297 (0.395) | 0.731 | -0.565 (0.216) | 0.024 | 1.084 (0.642) | 0.207 |
| TikTok Trend vs. Others | -0.484 (0.356) | 0.359 | -0.015 (0.194) | 0.997 | -0.820 (0.585) | 0.336 |
| TikTok Trend vs. Education | -0.188 (0.301) | 0.804 | 0.550 (0.163) | 0.002 | -1.904 (0.489) | <.001 |
